# Tuning Ternary Deep Eutectic Solvent Semiconductivity and Specific Capacitance Properties via Solubilizing Bacterial Nanocellulose for Flexible Soft Material

**DOI:** 10.1021/acsmaterialsau.5c00063

**Published:** 2025-09-16

**Authors:** Maurelio Cabo, Samir Kattel, Dennis LaJeunesse

**Affiliations:** † Department of Nanoscience, Joint School of Nanoscience and Nanoengineering, 14616 University of North Carolina Greensboro, Greensboro, North Carolina 27455, United States; ‡ Department of Applied Science and Technology, 3616 North Carolina Agricultural and Technical State University, Greensboro, North Carolina 27411, United States

**Keywords:** Bacterial Nanocellulose, Deep Eutectic Solvent, Tannic Acid, Solubility, Semiconductor, Flexible Materials

## Abstract

Bacterial nanocellulose (BNC) shows promise in sustainable materials science, but its insolubility limits broader applications. This study introduces a ternary deep eutectic solvent (TDES) composed of Choline Chloride, Imidazole, and Tannic acid to effectively dissolve BNC. The resulting solution exhibits enhanced bandgap energy, increasing from 4.348 to 4.528 eV (direct) and 4.156 to 4.471 eV (indirect), highlighting its potential application in a wide-bandgap semiconductor. Cyclic voltammetry revealed improved specific capacitance, indicating enhanced energy storage capacity. Its application in flexible soft material underscores its viability as a highly insulating yet sufficiently conductive material for future studies in biosensors, optoelectronics, and solar cells. By overcoming BNC’s solubility challenges while enhancing TDES properties, this study advances biobased electronics and optical applications, paving the way for eco-friendly technological innovations.

## Graphical abstract

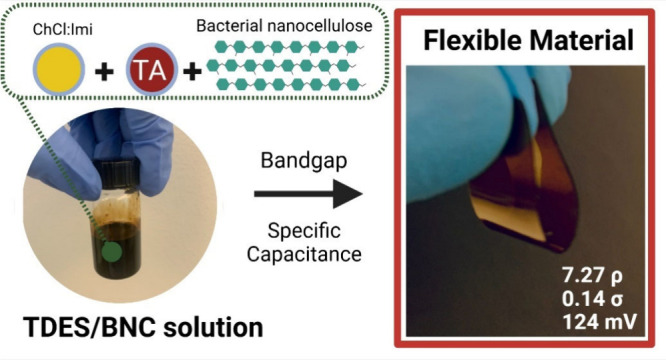

Bacterial nanocellulose (BNC) consists of long, high-molecular-mass polymer chains that are hydrogen-bonded, resulting in a highly stable structure making it an attractive material for a wide range of advanced applications.^1,2^ However, like other types of cellulose, BNC is insoluble in water and most organic solvents,^3^ which poses a significant challenge for its processing and functionalization. Several methods for solubilizing BNC have been explored to address this challenge. Lindman et al.^4^ examine a variety of strategies for thoroughly solubilizing BNC. Traditional approaches include the use of solvents such as lithium chloride/N,N-dimethylacetamide,^5^ NaOH/urea aqueous solutions,^6^ ZnCl_2_ aqueous solutions,^7^ ionic liquids (ILs),^8^ and *N*-methylmorpholine-N-oxide (NMMO) hydrate.^9^ While these methods have proven to be effective to varying degrees, they often come with limitations. For example, ionic liquids have garnered considerable interest due to their beneficial properties, such as thermal stability and low vapor pressure, making them attractive alternatives to harmful organic solvents in various industrial applications, including energy storage and biomaterial treatment.^10^ However, recent research has raised concerns about the toxicity of many ionic liquids, which poses new challenges in the functionalization of nanocellulose for environmentally sensitive applications.^11^ In response to these concerns, a new class of solvents known as deep eutectic solvents (DESs) has emerged as a promising alternative. DESs are formed by combining two or more safe, affordable, and renewable components, resulting in a eutectic mixture with a lower melting point than those of its individual components.^12^

The use of cellulose, especially BNC, to reduce the environmental impact from bioelectronics and semiconductor applications is a relatively new research area. Studies by Ramezani et al. demonstrated the potential of cellulose-based materials in flexible and transparent electronic devices, underscoring their promise for the semiconductor industry.^13^ Although DES-modified cellulose derivatives have shown potential in creating biobased conductive materials, applying DES to dissolve and functionalize BNC for semiconductor purposes represents a new and emerging avenue.^14^ Herein, we introduce a novel approach using a DES reinforced with tannic acid, creating a ternary deep eutectic solvent (TDES), to dissolve highly pure bacterial nanocellulose. A design of the experiment using the Taguchi method allowed us to fine-tune conditions for BNC dissolution in TDES. The result is a wider bandgap energy and improved capacitance. Controlling BNC dissolution and functionalization in this solvent system opens possibilities for flexible applications in which tunable properties are crucial.

Bacterial nanocellulose (BNC) was fabricated using the *Gluconacetobacter hansenii* strain using Hestrin–Schramm media for 14 days, Figure S1­(A). Before dissolution, BNC in dried form was characterized to measure its purity, crystallinity and morphology by using FTIR, XRD (Figure S4 and Table S3), and SEM (Figure S5). The ternary deep eutectic solvent (TDES) system was synthesized using choline chloride and imidazole in a 3:7 molar ratio with 0.5% (w/v) tannic acid, Figure S1­(B). TDES exhibited a yellowish color that transformed into a reddish black shade upon addition of tannic acid when subjected to 120 °C heat for 3 h. This color could indicate shifts in the electronic structure.^15^ The melting point temperatures of DES and TDES were measured using DSC, Figure S6, and comparable with those of the previous study by Zdanowicz et al.^16^ The dissolution of bacterial nanocellulose into TDES was utilized by using an L_9_3^4^ array, the Taguchi method Design of Experiments (DOE),^17^ herein, and four variables -- drying procedure, NaOH treatment concentration, BNC content % (w/v), and temperature setting -- at three levels as listed in Tables S1 and S2.

Out of the 9 experiments, five (E3, E4, E6, E7, and E8) successfully dissolved the BNC into the TDES solution, Figure S2­(A-B), wherein our analysis revealed that temperature had a most likely significant impact on the successful dissolution of BNC, Figure S2­(C). Picchio et al.^18^ demonstrated the formation of a natural deep eutectic solvent from choline chloride and tannic acid, indicating that tannic acid in the choline chloride–imidazole system acts as a cosolvent to facilitate BNC dissolution. For further confirmation, these runs were assessed by using a cellulose regeneration assay in water,^19^ Figure S3, wherein a clear separation with water means nondissolution of BNC into TDES, while a homogeneous solution shows successful solubility of BNC into TDES.

We focused our further characterizations in terms of band gap and specific capacitance on successful runs. For easier following, the samples with dissolved BNC are renamed into TDES/BNC_1 for E3, TDES/BNC _2 for E4, TDES/BNC _3 for E6, TDES/BNC _4 for E7 and TDES/BNC _5 for E8, see Table 1. For controls, we used the DES and TDES samples’ nomenclature.

**Table 1:** Experiments with Dissolved BNC from the Taguchi Method

	Pre-Dissolution		During Dissolution		After Dissolution	
Sample Name	Drying Technique (A)	Alkaline Treatment Concentration (B)	%w/v (C)	Set Temperature (D)	Viscosity (Pa·s)	Absorbed Stress (dyn/cm^2^)
TDES/BNC_1	OD	0.3 M	1.5%	180 °C	0.13 ± 0.10	39.18 ± 10.36
TDES/BNC_2	FD	0.1 M	1.0%	180 °C	0.10 ± 0.10	29.62 ± 5.99
TDES/BNC_3	FD	0.3 M	0.5%	160 °C	0.29 ± 0.30	75.58 ± 10.55
TDES/BNC_4	MD	0.1 M	1.5%	160 °C	0.19 ± 0.18	53.72 ± 9.45
TDES/BNC_5	MD	0.2 M	0.5%	180 °C	0.08 ± 0.08	24.91 ± 6.0

We used UV–vis spectroscopy to quantify the optical bandgap energy, Figure 1(A), using the Tauc method. The semiconductor band gap is the minimum energy difference between the highest valence band and the lowest conduction band. There are two categories: (i) direct, when the crystal momentum (k) of both bands is identical, and (ii) indirect, when k differs.^20^ Previous studies show that the eutectic system’s composition influences these energy levels.^21^ Abbott et al. further reported that adding hydrogen-bond donors (HBD) can transform a eutectic system into an ionic solution, shifting charge transport from holes to ions.^22^ Our results show that introducing bacterial nanocellulose significantly altered the bandgap of the TDES system, influenced by the BNC drying technique, NaOH treatment, BNC % (w/v), and dissolution temperature. Compared to controls—DES, 4.156 eV (indirect) and 4.348 eV (direct), and TDES, 4.463 eV (indirect) and 4.519 eV (direct)—all BNC-loaded samples exhibited modified bandgap values, confirming that BNC integration tunes optical properties, Figure 1(B).

**Figure 1:**
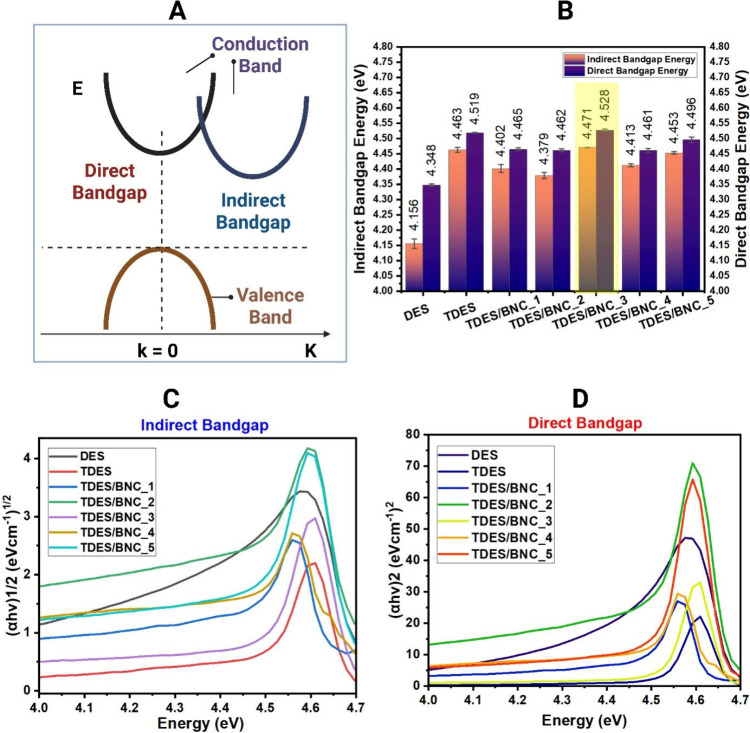
Direct bandgap energy allows an electron to emit a photon without changing its momentum, while indirect bandgap energy involves an intermediate state (A). All samples generate both direct and indirect bandgap energy (B). Using UV–vis, the shift from left to right or lower to higher bandgap indicates the capacity of solubilized bacterial nanocellulose to tune ternary deep eutectic solvent indirect bandgap (C) and direct bandgap (D).

Among all, TDES/BNC_3 displayed the highest bandgap (4.471 eV for indirect, 4.528 eV for direct), attributed to its set parameters: freeze-dried BNC, BNC alkaline treatment at 0.3 M, BNC added content at 0.5%, and dissolution temperature at 160 °C. This condition most likely preserved the BNC nanofibrillar structure, see Figure S7­(C), while promoting sufficient interfacial interaction with TDES, resulting in enhanced optical properties. Correspondingly, this sample also exhibited the highest viscosity, 0.29 Pa·s, and absorbed stress at 75.58 dyn/cm^2^, indicating an entangled nanofiber network that contributed to the observed increase in both direct and indirect bandgap values.^23^ In contrast, TDES/BNC_1 and TDES/BNC_2—processed at 180 °C with higher BNC content—showed slightly reduced bandgap values compared to those of TDES. Specifically, TDES/BNC_1, which used oven-dried BNC with 1.5% loading and 0.3 M NaOH concentration for the pretreatment process, may have experienced structural aggregation, see Figure S7­(A), lowering the bandgap to 4.402 eV (indirect) and 4.465 eV (direct), and it also showed relatively low viscosity at 0.13 Pa·s and absorbed stress at 39.18 dyn/cm^2^.^24^ Meanwhile, TDES/BNC_2, although it dissolved freeze-dried BNC and used a lower alkaline concentration for BNC pretreatment, 0.1 M, showed slightly lower bandgap values, 4.379 eV indirect and 4.462 eV direct, suggesting sufficient nanofiber dispersion; see Figure, S7­(B). Its lower viscosity, 0.10 Pa·s, and stress, 29.62 dyn/cm^2^, also support a less interconnected structure.^25^ TDES/BNC_4 with 4.413 eV (indirect) and 4.461 eV (direct) and TDES/BNC_5 with 4.453 eV (indirect) and 4.496 eV (direct) offered intermediate bandgap values, yet both used a microwave-drying technique for BNC. Notably, TDES/BNC_5, with other dissolution parameters set at 0.2 M NaOH, 0.5% (w/v), and 180 °C, achieved higher bandgap compared to TDES/BNC_4, indicating the benefits of lower BNC loading with elevated set temperature and BNC alkaline concentration pretreatment. In addition, despite having the lowest viscosity, 0.08 Pa·s, and absorbed stress, 24.91 dyn/cm^2^, this suggests that low-concentration BNC can still influence electronic behavior, possibly through enhanced molecular alignment and less interchain entanglement, see Figure S7­(D) and Figure S7­(E).^26^ This study highlights that the combination of dissolved BNC content percentage, alkaline pretreatment concentration, dissolution set temperatures, and BNC drying techniques can effectively tune the bandgap energy. This underscores the importance of structural tuning to advance biobased semiconductors for optoelectronic applications. The UV–vis spectra showing the shift from lower to higher bandgap are presented in Figure 1(C) for the indirect bandgap and in Figure 1(D) for the direct bandgap. All sample measurements for both indirect and direct bandgaps are provided in Figure S8. To ensure unbiased results, the analysis was performed using the Linear Quickfit function in OriginPro software, which automatically adjusted the slope of the fitted line.

We further suggest that this tunable semiconductivity was caused by the movement of ions,^27^ which facilitates the direct and indirect transition of electrons from the valence band to the conduction band by the absorption of thermal energy. In addition, ions generate an electric field that facilitates electronic motion, influencing the energy required for their transition to the conduction band. In our system, the loose bonding between atoms in the DES allows ions to flow freely, enhancing charge transfer and semiconductivity.^28^ Unlike direct bandgap semiconductors, indirect bandgap materials do not allow electrons to transition directly from the valence band to the conduction band only by absorption of light or heat; instead, these materials require an adjacent ion to modify their energy level before transitioning.^29^ DESs enable ions to engage with electrons upon exposure to higher temperature, facilitating the dissolution and unrestricted movement of ions and changing the character of the semiconductor electronic properties.^30,31^ Likewise, in Figure S9­(A), the increase in ionic conductivity upon the introduction of tannic acid and the dissolution of bacterial nanocellulose indicates enhanced ion mobility; notably, the TDES/BNC_1–4 samples required lower concentrations to achieve higher conductivity. In Figure S9­(B), the FTIR spectra provide additional insights, showing shifts in the carbonyl stretching peak (C=O, ν) from 1600 to 1720 cm^–1^ and band broadening in the O–H stretching region (3500–2250 cm^–1^).^18^

To examine the electrochemical behavior of the samples, cyclic voltammetry was performed at scan rates of 60, 80, and 100 mV/s. For each test, 10 mL of sample solution was transferred in the electrochemical cell, into which three probes were immersed; see the setup in Figure S10. The choice of different scan rates allows us to observe how the system responds to varying electron transfer speeds. At lower scan rates (60 mV/s), Figure 2(A-C), the peaks are sharper and more defined, indicating that the system has sufficient time to equilibrate. As the scan rate increases to 80 (Figure 2(D-F)) and 100 mV/s (Figure 2(G-I)), the peaks become broader and shift, reflecting the faster electron transfer and diffusion limitations at higher scan speeds. From the potential range of −1 to 1.5 mA, there are slight to nonsignificant oxidation or reduction peaks for DES even when tannic acid was added, indicating the possible absence of any electron transfer within the electrolyte.^32^ The absence of any redox peaks also demonstrates the non-faradaic interaction between the samples and electrodes. However, the addition of bacterial nanocellulose shows a significant change in oxidation and reduction peaks, as shown in the much closer look in Figure 2(B,E,H). It is also evident when there is a tremendous increase in the specific capacitance as shown in Figure 2(C,F,I), which means the DES and TDES energy storage capacity improved due to the presence of nanofibers from bacterial nanocellulose. This also means that the complex structure of bacterial nanocellulose, which includes the linked structure, may spread the electroactive substances to the surface of the electrode, which can change the total area of the cyclic voltammogram.^33^

**Figure 2:**
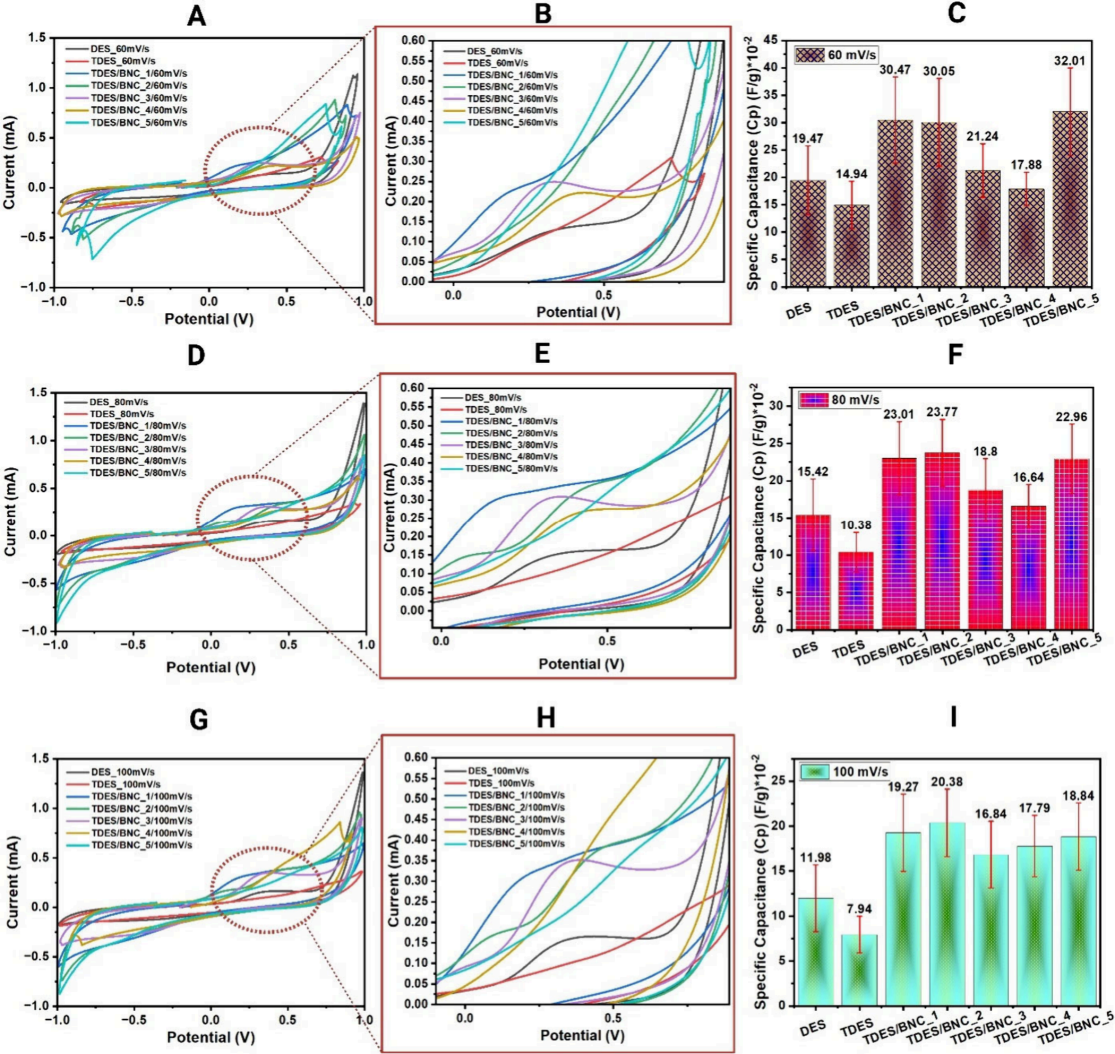
Cyclic voltammetry measurements were performed in triplicate on three cycles using various scan rate conditions: 60 mV/s (A, B, C), 80 mV/s (D, E, F), and 100 mV/s (G, H, I).

For further discussion, based on the results, the specific capacitance across varying scan rates reveals how each processing factor — drying method, alkaline treatment, BNC concentration, and dissolution temperature — affects the electrochemical performance of the TDES/BNC solution and how this behavior is interconnected with their semiconductivity, viscosity, and absorbed stress. Table S4 presents the area of integration calculated to determine the specific capacitance.

At 60 mV/s, four out of five TDES/BNC solutions significantly outperformed DES, 19.47 × 10^–2^ F/g, and all TDES/BNC solutions outperformed TDES, 14.94 × 10^–2^ F/g, indicating that incorporating BNC enhances the charge storage capacity. TDES/BNC_5 exhibited the highest specific capacitance, 32.01 × 10^–2^ F/g, followed by TDES/BNC_1, 30.47 × 10^–2^ F/g, TDES/BNC_2, 30.05 × 10^–2^ F/g, TDES/BNC_3, 21.24 × 10^–2^ F/g, and TDES/BNC_4, 17.88 × 10^–2^ F/g. The superior performance of TDES/BNC_5, which used microwave-dried (MD) BNC, alkaline treatment at 0.2 M, and BNC content at only 0.5%, suggests a potential balance between electrical conductivity and ionic transport.^34^ Despite having the lowest viscosity and absorbed stress, this sample further possibly allowed enhanced ion mobility, leading to greater double-layer formation and redox interactions—both key contributors to a higher capacitance. Conversely, TDES/BNC_3, despite its highest bandgap and rheological properties, and TDES/BNC_4 showed a lower capacitance, which highlights a potential trade-off that while better network structuring improves the semiconductive property and optical bandgap, it may limit ion accessibility and hinder capacitance.^35^ This inverse relation underlines the need to balance structural rigidity with electrochemical accessibility in biobased materials. TDES/BNC_1 and TDES/BNC_2 both achieved high capacitance values, with TDES/BNC_1 slightly outperforming TDES/BNC_2 due to the oven-drying (OD) technique and higher NaOH BNC treatment concentration, which might better preserve the bulk nanostructure and enhance electrolyte accessibility. At 80 and 100 mV/s, specific capacitance values decreased as expected due to reduced ion diffusion. Interestingly, TDES/BNC_4 gained a comparative edge at 100 mV/s, 17.79 × 10^–2^ F/g, suggesting that higher BNC content at 1.5 (%w/v) may contribute to better performance at faster scan rates, likely due to increased surface area for charge accumulation.^36^ The data suggest that specific capacitance is influenced by how well the system balances ionic accessibility, favored by lower viscosity and stress, with structural support through enhancing semiconductivity and bandgap.

Incorporating DES, TDES, and TDES/BNC into agar/glycerol solutions produced flexible semiconducting materials. TDES/BNC_3, selected for its highest bandgap, demonstrated excellent flexibility during bending, twisting, stretching, and rolling, Figure 3(A). Tensile testing, Figure 3(B), confirmed fracture behavior, while DMA, Figure 3(C), measured storage and loss moduli and tan δ at 25–40 °C. In Figure 3(D), TDES/BNC_flex achieved the highest tensile strength, 3.68 N/mm^2^, a 10.51% increase over DES_flex and 23.91% over TDES_flex, with slightly higher strain. Stress–strain curves and Load vs Displacement are shown in Figure S11. DMA results, Figure 3(E), revealed a decreasing storage modulus trend, with TDES/BNC_flex exhibiting the lowest storage, 8.03 GPa, and loss modulus, 0.35 GPa. This indicates that, despite higher strength, the material remains highly flexible with minimal energy dissipation, making it efficient for potential applications in wearable and semiconducting devices. Tan δ values, Figure 3(F), below 1 confirm elastic behavior.

**Figure 3:**
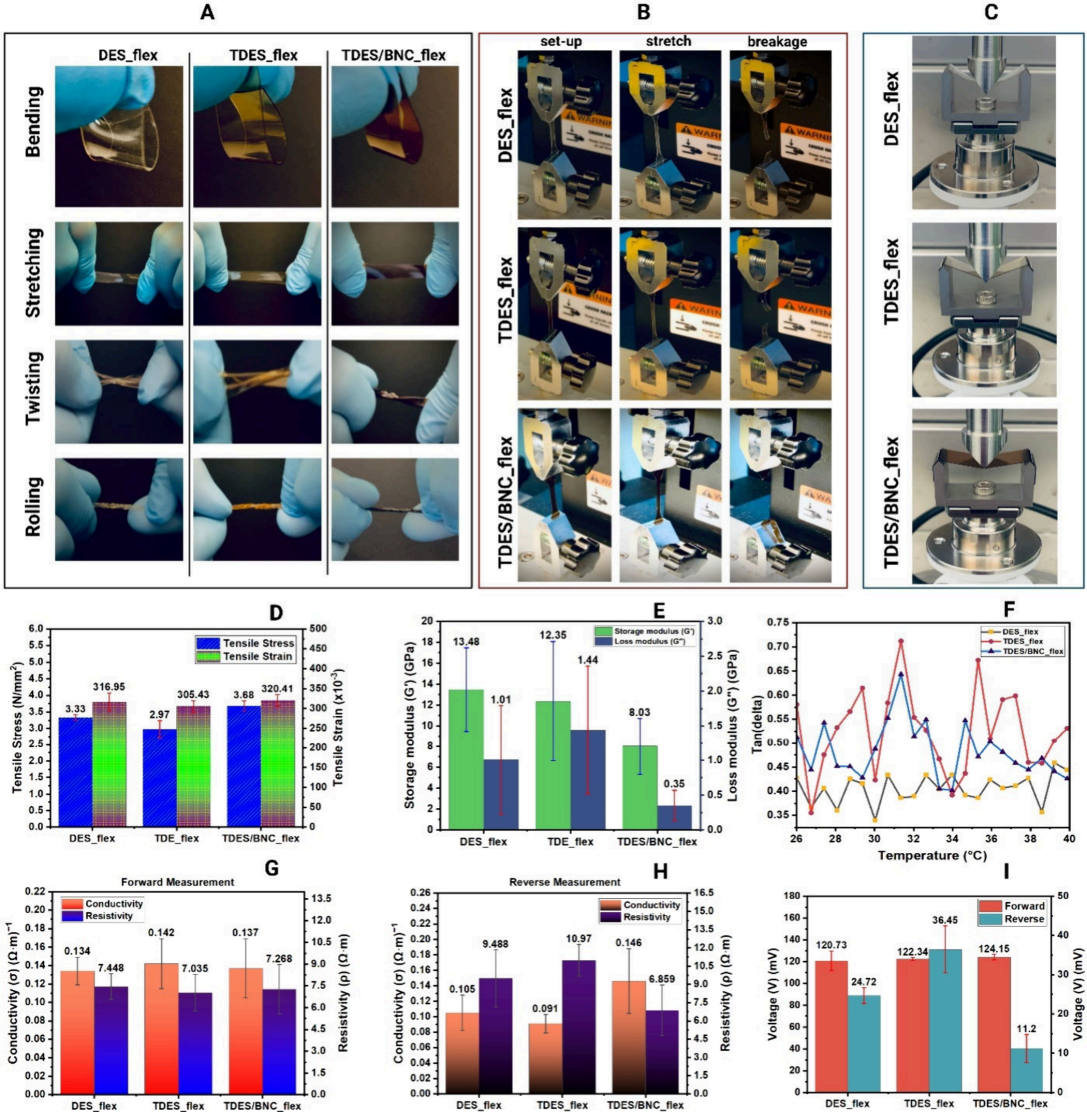
Flexibility performance (A); experimental setups for tensile testing (B) and DMA (C); results for tensile stress and strain (D), storage and loss modulus (E), tan­(δ) (F), electrical conductivity and resistivity (G–H), and voltage drop (I) of DES, TDES, and TDES/BNC flexible soft materials.

A four-point probe (5 μA) measured the resistivity and conductivity. All samples showed higher resistivity than conductivity in both forward, Figure 3(G), and reverse modes, Figure 3(H), with resistivity values comparable to silicon semiconducting properties, 0.1–2300 (Ω·m).^37^ Voltage generation trends, Figure 3(I), mirrored conductivity and resistivity, suggesting that BNC introduces electrostatic interactions with choline chloride, imidazole, and tannic acid, enabling stored resistance, conductivity, and voltage output.

This study demonstrates that a tannic acid-reinforced deep eutectic solvent (TDES) effectively dissolves bacterial nanocellulose (BNC), overcoming its inherent insolubility. The Taguchi design revealed that the drying method, NaOH treatment, BNC content, and temperature critically influence dissolution efficiency. Incorporating BNC into TDES enhanced the optical properties, increasing both direct and indirect bandgap energies vital for biosemiconductors. Cyclic voltammetry confirmed a sufficient specific capacitance for energy storage. In addition, TDES/BNC exhibited electrical performance within the range of semiconducting materials, supporting its potential as a sustainable, high-performance biomaterial for flexible electronics, wearable sensors, and bioelectronic devices. While this work focuses on dissolution efficiency and functional performance, future studies should investigate the sustainability of TDES/BNC, including recyclability, postuse recovery, and biodegradability, to advance its development as an ecocompatible platform for next-generation flexible devices.

## Supplementary Material



## Data Availability

Data available on request from the authors.

## Notes

Additional experimental details; Characterization; Statistical Analysis; The Taguchi Method – Reaction Conditions (DOE); Weighted masses used for Bacterial nanocellulose dissolution; % Purity, CrS, and % Crystallinity of Alkaline Treated Bacterial Nanocellulose; Average of CV area of Integration to measure specific capacitance; Cellulose Regeneration Assay in Water; SEM images of alkaline-treated dried nanofibers from bacterial nanocellulose; Melting temperature of the DES and TDES; TEM images showing BNC structure in TDES after dissolution; Measured Indirect and direct band in triplicate samples; Ionic Conductivity and Ionic Concentration and ATR-FTIR spectrum; Cyclic Voltammetry set up; Stress–Strain curve and Load vs Displacement of flexible soft materials (PDF)
