# Supplementary material for: Tuning Ternary Deep Eutectic Solvent Semiconductivity and Specific Capacitance Properties via Solubilizing Bacterial Nanocellulose for Flexible Soft Material
Source: ACS Mater Au. 2025 Sep 16;5(6):971–7. doi: 10.1021/acsmaterialsau.5c00063 (PMC12616433; doi:10.1021/acsmaterialsau.5c00063)
Supplement: Supplementary file 1 [file mg5c00063_si_001.pdf]

Supplementary Information

Tuning Ternary Deep Eutectic Solvent Semiconductivity and Specific Capacitance Properties via Solubilizing Bacterial Nanocellulose for Flexible Soft Material

Maurelio Cabo Jr.,[1] Samir Kattel,[1,2] Dennis LaJeunesse [1,*]

[1]Department of Nanoscience, Joint School of Nanoscience and Nanoengineering, University of North Carolina Greensboro, Greensboro, North Carolina, 27455, USA
[2]Department of Applied Science and Technology, North Carolina Agricultural and Technical State University, Greensboro, North Carolina, 27411, USA

[*] Corresponding author. *drlajeun@uncg.edu**

A. Experimental Sections

1. Materials:

Dextrose, Yeast extract, NaOH pellets, Na_2HPO_4, Agar powder, Peptone, Tannic Acid, Choline Chloride (99%), Glycerol and Dimethylformamide (DMF) were purchased from Fisher Scientific (Thermo Fisher Scientific, Waltham, MA, USA). Imidazole (99%) and Citric Acid were purchased from Sigma-Aldrich (Sigma-Aldrich, St. Louis, MO, United States). Pellicles of BNC were collected from cultures of *Gluconacetobacter hansenii* (ATCC 23769, American Type Culture Collection, Manassas, VA, USA).

2. **Culturing and Fabrication of Bacterial Nanocellulose**

Gluconacetobacter hansenii was cultured in Hestrin-Schramm (HS) media composed of 2% (w/v) Dextrose, 0.5% (w/v) yeast extract, 0.5% (w/v) peptone, 0.27% (w/v) Na_2HPO_4, and 0.125% (w/v) citric acid. To inoculate the bacterial strain, an agar plate (85 mm × 15 mm) with the same HS media composition and 2% agar was prepared and incubated at 30°C for 2 days. A pea-sized bacterial colony was then transferred from the inoculation plate into 1000 mL of media in a 9” × 13” (3-quart) Anchor Hocking glass baking dish and cultured for 14 days at 25°C. After the incubation period, the pellicles were treated in a selected NaOH concentration at 95°C for 1 hour to remove bacterial cells and biofilm materials, leaving only the bacterial nanocellulose matrix. The pellicles were then washed with distilled water until a neutral pH was reached and stored in deionized (DI) water at room temperature. See **Fig. S1(A)** for illustration.

3. **Design of Experiment**

In Taguchi method, $L_9 3^4$ array Design of Experiments (DOE) we incorporate four variables at three different levels – L1, L2, L3 (refer to **Tables S1)**. Oven-drying (OD) was employed using oven dryer set at 70^0C. Freeze-Drying (FD) was done using Labconco FreeZone Triad Freeze Dryer, a three-in-one lyophilizer set at the temperature of -85^0C. Microwave-Drying (MD) employed using a typical kitchen model which set at 100^0C.

4. **Synthesis of Deep Eutectic Solvent and Dissolution of Bacterial Nanocellulose**

In 3:7 molar ratio **[1]**, we mixed choline chloride (8.39 g) and imidazole (9.53 g) and heat up at 120ºC in a hotplate. The mixture turned into yellowish liquid solution and let it rest for 24 h in room temperature. An amount of tannic acid, **see Table S2** was added in the following day and the liquid color transformed into a reddish black shade, **Fig. S2(A).** Weighted masses used to solubilize bacterial nanocellulose was disclosed in **Table S2.** After dissolution into the TDES solution, **Fig. S2(B),** the color of the mixture changed into strong black, and viscosity increased, **Table S1**. We used MATLAB to analyze the factor interactions influencing both the dissolution and non-dissolution of BNC in the TDES solution, **Fig. S2(C) [2].** To find out which experiments successfully dissolve BNC into TDES, we perform a cellulose regeneration test in water and then spin it in a centrifuge **(Fig. S3).**

5. Flexible Soft Material Fabrication

In 250 mL of DI water, 5 g of agar and 15 g of glycerol were mixed and autoclaved at 115°C for 45 minutes **[3]**. While hot, 300 µL of DES, TDES, and TDES/BNC were each added to 20 mL of the agar-glycerol solution and poured slowly into an 85 mm × 15 mm petri dish. The solution was left to dry at room temperature for three days before the thin film was removed from the petri dish. The film was cut into 40 x 10 mm (L x W) size for testing.

B. Characterization

Before dissolution, all alkaline treated bacterial nanocellulose were characterized using FTIR, SEM, and XRD to determine its purity, crystallinity, and nanofiber morphology and sizes, see **Fig. S4 and Fig. S5** and **Table S3.** To quantify BNC purity, IR spectra were recorded on a Fourier-transform infrared spectroscopy (FTIR) spectrometer (Agilent 670 FTIR Spectrometer, Santa Clara, CA, USA) under dry air at ambient temperature. The percentage of transmittance spectra was recorded from 4000 to 400 cm^{-1} with 64 scans in each case at a resolution of 4 cm^{-1}. For purity proportional percentage calculation of alkaline treated bacterial nanocellulose, the following equation was used based on the Yamamoto et al mass fractions equation [**4**]:

$$\%Purity = \frac{A_{Cellulose}Peaks}{A_{CellulosePeaks} + A_{ImpuritiesPeaks}} \text{ x } 100 \qquad (1)$$

The peak designations for Area of cellulose peaks ($A_{cellulose\ peaks}$) and Area of impurities peaks ($A_{impurities\ peaks}$) were based on the previous study conducted by Grube et al wherein for the first time FT-IR spectra of different origin BNC samples were recorded and analyzed to select the best method for screening and/or evaluation of the BNC quality [**5**].

Crystallinity analysis was conducted using Rigaku SmartLab equipment with Cu-Kα radiation. A copper source was used at 40 V and 40 mA. The samples were mounted on the sample holder, and the patterns were recorded by running the instrument at a speed of 5°/ min and a 2θ range of 5°–40°. The Scherrer's formula was used to measure the crystallite size:

$$CrystalliteSize = \frac{k\lambda}{W} cos\theta \qquad (2)$$

(with a shape factor k = 0.94 was employed to determine the crystallite sizes of samples with full width at half maximum (fwhms, W) and peak centers obtained by fitting the (110) and (200) peaks at the Gaussian function using OriginPro software; here λ is the wavelength of X-ray radiation (0.154 nm) [**6**].

The crystalline index (CrI^{XRD}) was calculated with the help of the following formula:

$$CrI = \left(\frac{I_{200}-I_{am}}{I_{200}}\right) x100 \quad (3)$$

Where I_{200} is the maximum intensity of the (200) lattice diffraction and I_{am} is the intensity diffraction at 16° (2θ) [**7-8**].

The melting temperatures were measured via DSC technique. Samples were weighed into aluminum pans (10-20 mg), sealed, and analyzed with modulated runs in the temperature range from −60 to 200°C, with an isothermal run over the first 2 mins at 50°C and then with a heating rate of 5°C/min in a nitrogen atmosphere and a second isothermal run in 15 mins at 15°C.

The TEM images of bacterial cellulose samples were investigated using JEOL 2100PLUS high-resolution transmission electron microscopy (HR-TEM) operated at an accelerating voltage of 200 kV. To prepare the sample, 10 µL was mixed with 600 µL of 70% ethanol in Eppendorf tubes (0.6 mL), 1:100 serial dilution, then it was sonicated and placed on a carbon-coated copper TEM grid for analysis.

UV-VIS analysis was employed by Thermo Scientific NANODROP 2000C. The samples were analysed in a 10 x 45 mm, 3.5mL quartz cuvette. The direct and indirect band gaps were measured in according to the UV-VIS-based Tauc method **[9].**

$$(\alpha \cdot hv)^{1/\gamma} = B(hv - E_g) \quad (4)$$

where h is the Planck constant, ν is the photon's frequency, Eg is the band gap energy, and B is a constant. The γ factor depends on the nature of the electron transition and is equal to 1/2 or 2 for the direct and indirect transition band gaps, respectively. To lower the concentration of all samples, DMF was used as a solvent to measure the absorbance. Here, 80µL of DES and 80µL of TDES was added to 5mL of DMF in a two separate test tubes. For TDES/BNC samples, 40µL was added to 5mL of DMF in each test tube. To ensure its miscibility, Vortex was used for mixing. The absorbance limit of detection was set from 0 to 1.0 a.u. All samples were run three times, and the average values was measured.

Ionic conductivity (0–20,000 µS/cm) and ionic concentration (0–18,000 mg/L) were measured using a NeuLog conductivity logger sensor (NUL-215). The samples at 200µL volume were dropped on the sensor for measurement. The same ATR-FTIR method described above was also used to analyze changes in carbonyl (C=O) absorbance and the broadening of hydroxyl (O–H) groups in the prepared DES, TDES, and TDES/BNC samples.

The cyclic voltammetry measurements were performed using three probe method. Data were collected with VMP3 Bio-Logic multichannel potentiostat. Three electrodes consisting of working electrode (WE), reference electrode (RE) and counter electrode (CE) was immersed in the sample solution (10 ml) kept in electrochemical cell, see Fig. S9. The working electrode was a glassy carbon (GC) disk with a diameter of 3 mm, giving a geometric surface area of 0.071 cm^2. To ensure a clean and reproducible surface, the GC disk was polished before each experiment. Polishing was carried out step by step using alumina slurries of different particle sizes (1.0, 0.3, and 0.05 µm) on a polishing cloth, starting with the coarsest and finishing with the finest.

After polishing, the electrode was carefully rinsed with deionized (DI) water to remove any alumina particles left on the surface. Finally, it was dried with a gentle flow of nitrogen gas before being placed into the electrolyte solution. The counter electrode was a platinum (Pt) wire, 5 cm long and 0.5 mm in diameter. Platinum was selected because it is highly conductive and resistant to chemical reactions during electrochemical testing. To keep the Pt surface clean, the wire was treated with concentrated nitric acid (HNO_3) to remove contaminants and oxide layers and then rinsed thoroughly with DI water to remove any remaining acid. The reference electrode was a saturated silver/silver chloride (Ag/AgCl) electrode filled with 3.0 M KCl solution. This type of electrode was used because it provides stable and well-defined potential, which is essential for accurate CV measurements. CV with scan rates 60, 80, 100 mV/s of samples was measured in the potential window of -1V to 1V. The choice of 60 mV/s as our starting scan rate was based on the previous study by Mu et al. on polyaniline nanofibers **[10].** The specific capacitance, C_p,was calculated with the following equation **[11]**:

$$C_p = \frac{A}{2mk(V_2 - V_1)} \quad (5)$$

Here, k is the scan rate, m is the mass of the active material, A is the area of the CV curve, and (V_2-V_1) is a potential window.

Flexible material fabricated was characterized Jandel Multi-Height 4-point probe with RM3 Test Unit to measure sheet resistance in ohms/square (Ω/□) and voltage in mV. Sheet resistance was converted into Resistivity, ρ (Ω·m) **[12]** and Conductivity, σ $(\Omega\cdot m)^{-1}$ **[13]** using the below equations:

$$\rho = R_s\, x\, t \quad (6)$$

Where ρ = resistivity; $R_s = \text{sheet resistance}$; and t = sample thickness in meter. Then conductivity is the reciprocal of resistivity:

$$\sigma = \frac{1}{\rho} \quad (7)$$

For mechanical properties, dog bone shaped samples underwent tensile strength testing using a universal testing machine (Torbal -Poland) and was cut based on the mold from Dogbone stamp ISO 37 **[14]**. The thickness, length, and width of each strip was measured using a digital caliper. Subsequently, the samples were secured between two clamps with a gauge length of 50 mm and subjected to a force at 10 mm/min rate under ambient temperature (23 °C) until the sample broke. Maximum stress and elongation at break were determined using the data from the Force Manager software. Stress (MPa) was calculated as the ratio of loading force (expressed in Newtons, N) to the cross -sectional area of the sample (A). Strain (%) was calculated as $\Delta L/L_o \times 100\%$, where L_o represents the initial length and ΔL signifies the extension from the starting point or the recorded maximum displacement. The mechanical tests represent the average of three experiments ± standard deviation (SD).

For storage modulus, loss modulus, and tan (delta), the TA HR20 Dynamic Mechanical Analyzer (DMA) was employed at 3-point bending set-up with geometry of 25 mm length. Axial force for sample compression set at 1 N and minimum axial force limit at 0.1 N.

C. Statistical Analysis

Using Minitab, orthogonal design was used to determine suitable tolerances for the components of a certain experiment **[15].** All experiments were performed at least in triplicate. For orthogonal design analysis of experiments, MATLAB was used for factors interaction analysis **[16-18].** Histogram graphs, Mechanical Properties, FTIR, XRD, UV-Vis, Specific Capacitance, Resistivity, Conductivity, Voltage, Ionic Conductivity and Concentration, CV graphs were generated using OriginPro 2024b Academic Software.

Table S1: The Taguchi Method – Reaction Conditions (DOE)				
Sample Name	Pre-Dissolution		During Dissolution	
	Drying Technique (A)	Alkaline Treatment Concentration (B)	%w/v (C)	Set Temperature (D)
E1	OD	0.1 M	0.5%	120ºC
E2	OD	0.2 M	1.0%	160ºC
E3	OD	0.3 M	1.5%	180ºC
E4	FD	0.1 M	1.0%	180ºC
E5	FD	0.2 M	1.5%	120ºC
E6	FD	0.3 M	0.5%	160ºC
E7	MD	0.1 M	1.5%	160ºC
E8	MD	0.2 M	0.5%	180ºC
E9	MD	0.3 M	1.0%	120ºC

Note: Heating Time in 7 h

Table S2: Weighted masses used for Bacterial nanocellulose dissolution in Choline Chloride-Imidazole-Tannic Acid Deep Eutectic Solvent

Experiment No.	Choline Chloride (g)	Imidazole (g)	Weight of Tannic Acid Added (mg)	Weight of BNC Solubilized (mg)
E1			85.9	55.6
E2			86.3	144.7
E3			86.7	202.7
E4	8.39	9.53	86.8	138.5
E5			71.9	174.5
E6			76.7	63.3
E7			76.9	204.3
E8			82.8	65.6
E9			83.8	128.4

Note: Choline Chloride, Imidazole and Tannic were dissolved at 120ºC in 3 h.

Table S3: % Purity, CrS, and % Crystallinity of Alkaline Treated Bacterial Nanocellulose			
Experiment No.	FT-IR	XRD	
	%Purity	Ave. Crystallite Size	% Crystallinity
E1	83.77	5.29	93.10
E2	94.99	5.42	97.80
E3	95.57	6.35	97.94
E4	90.90	5.14	94.25
E5	87.44	4.85	92.16
E6	90.53	5.24	91.09
E7	87.16	5.13	95.35
E8	91.45	4.66	95.29
E9	93.46	5.41	93.04

Table S4: Average of CV area of Integration to measure specific capacitance			
	**Area of Integration**		
**Samples**	**60 mV/s**	**80 mV/s**	**100 mV/s**
**DES**	0.467 ± 0.15	0.494 ± 0.15	0.479 ± 0.15
**TDES**	0.359 ± 0.11	0.332 ± 0.09	0.318 ± 0.08
**TDES/BNC_1**	0.731 ± 0.19	0.736 ± 0.16	0.771 ± 0.17
**TDES/BNC_2**	0.721 ± 0.19	0.761 ± 0.14	0.815 ± 0.15
**TDES/BNC_3**	0.510 ± 0.12	0.602 ± 0.13	0..674 ± 0.15
**TDES/BNC_4**	0.429 ± 0.07	0.532 ± 0.09	0.711 ± 0.14
**TDES/BNC_5**	0.768 ± 0.19	0.735 ± 0.15	0.754 ± 0.15

Note: Sample mass used = 10 g; ΔV = 2

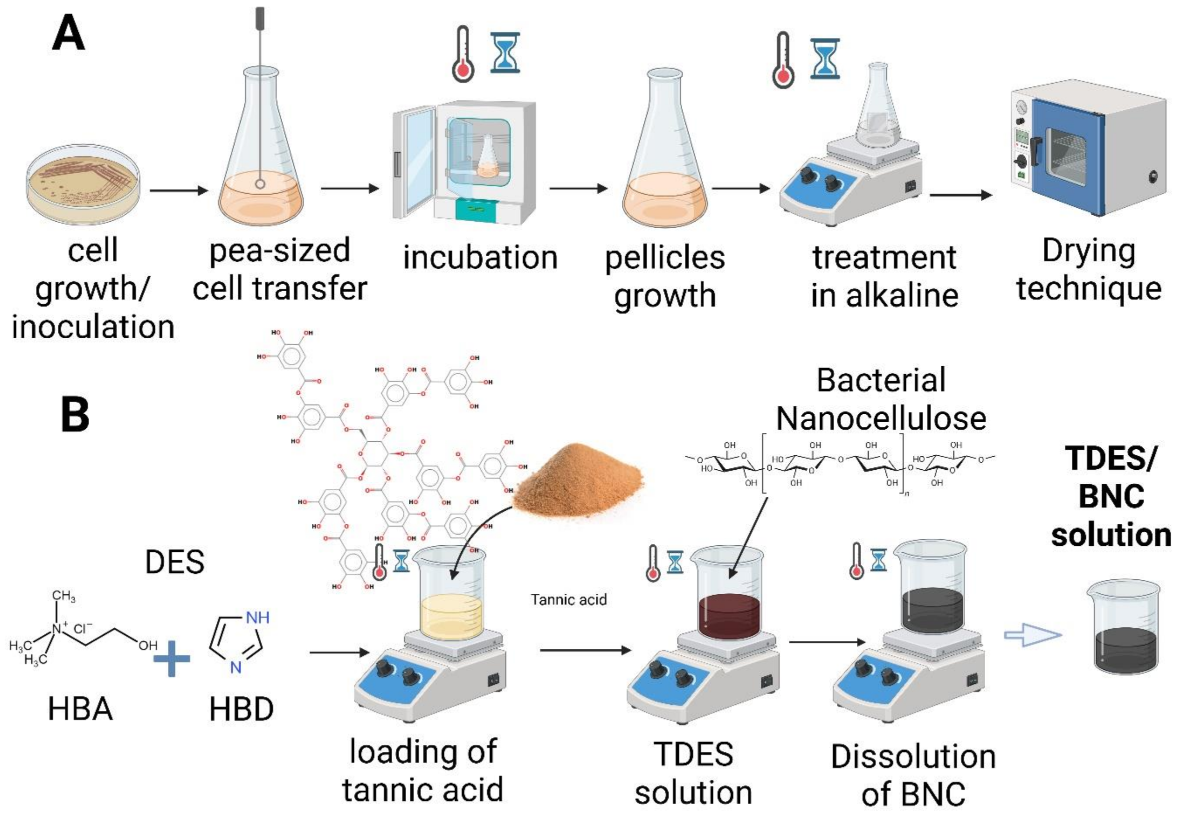

Figure S1. (A) Bacterial nanocellulose fabrication and (B) synthesis of choline chloride/imidazole (DES) as a deep eutectic solvent with tannic acid (TDES) and dissolution of BNC (TDES/BNC).

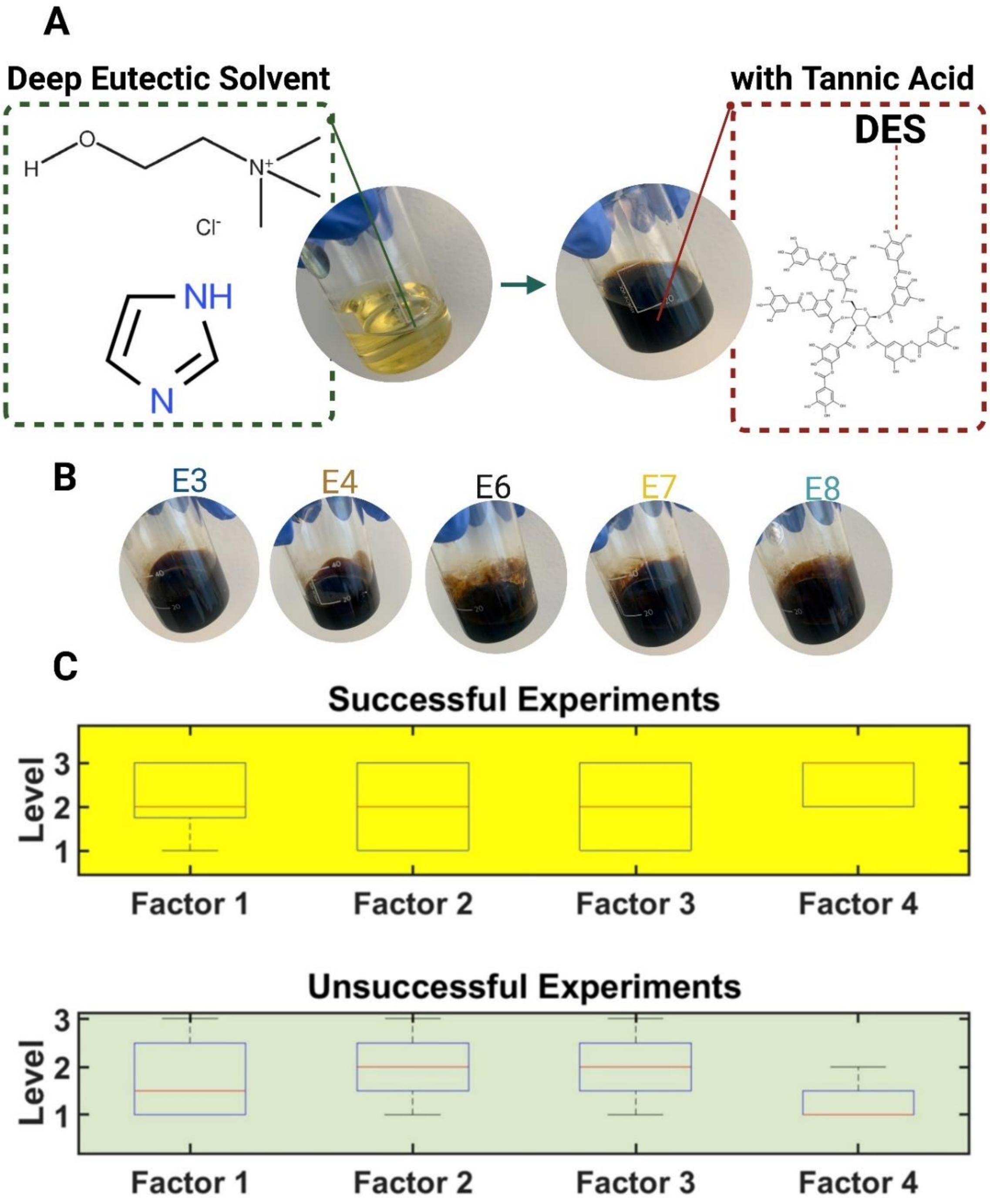

Figure S2: (A) The liquid solution of DES (right) and TDES (left); (B) The 5 experimental runs show the dissolved nanofibers from bacterial nanocellulose; (C) MATLAB was used to analyze the factor interactions influencing solubility of BNC into TDES.

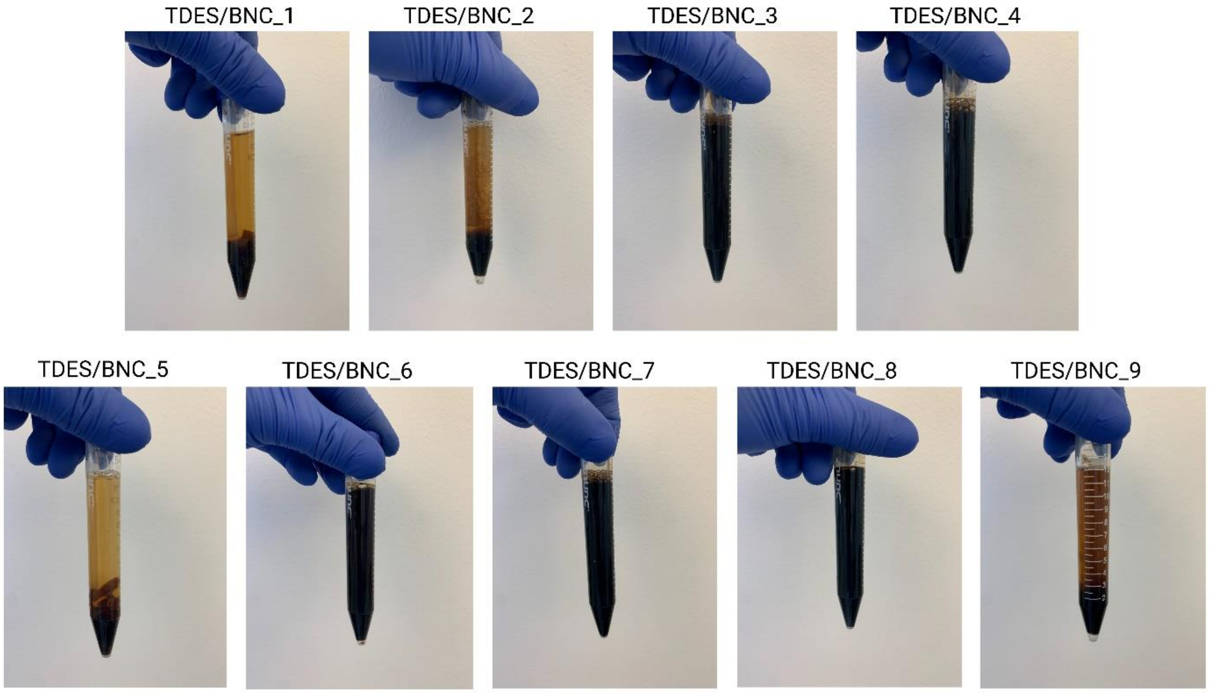

Figure S3: Cellulose Regeneration Assay in Water.

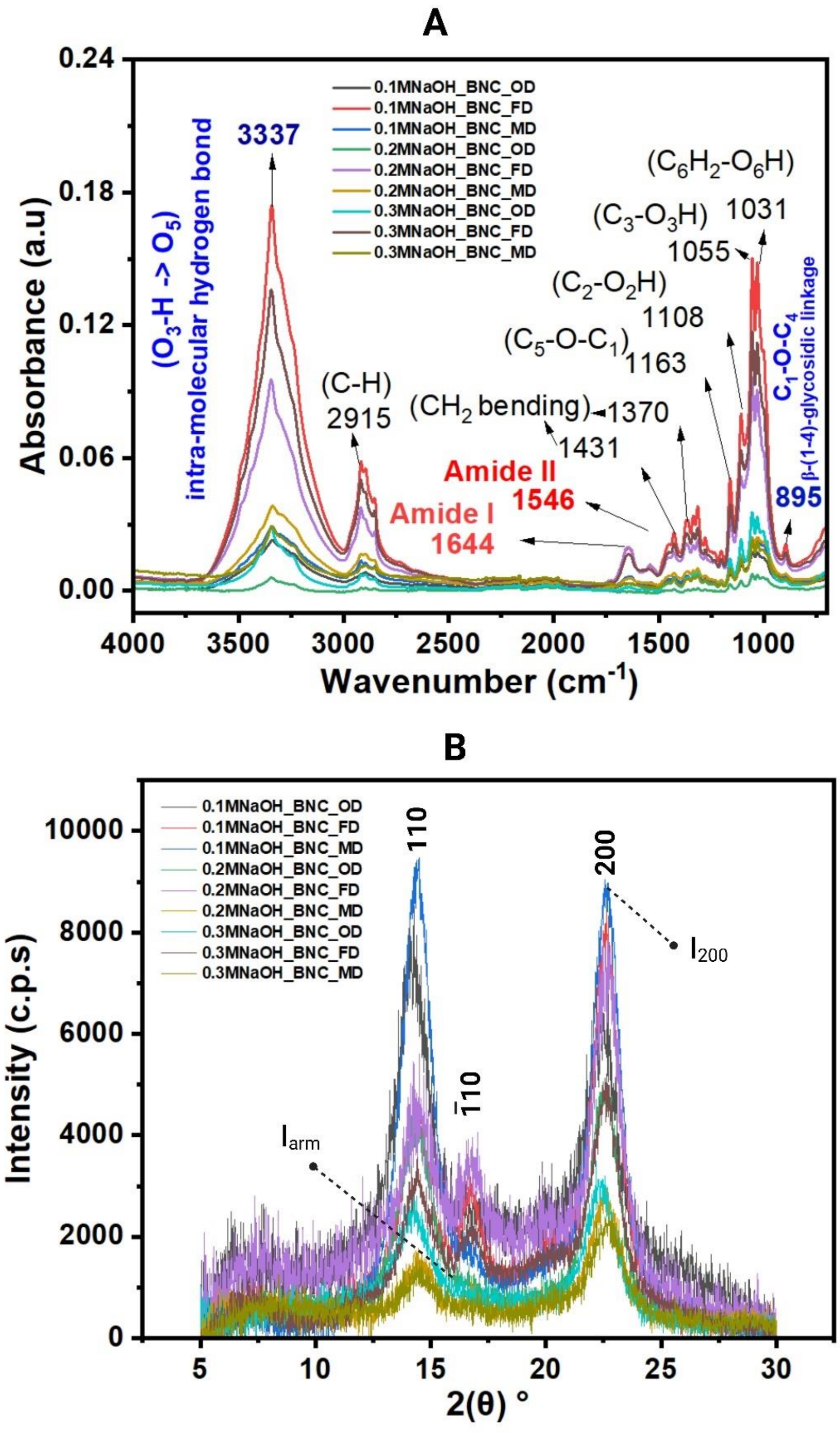

Figure. S4. Characterization of Dried Bacterial Nanocellulose in terms of purity using ATR-FTIR (A) and degree of crystallinity using XRD (B)

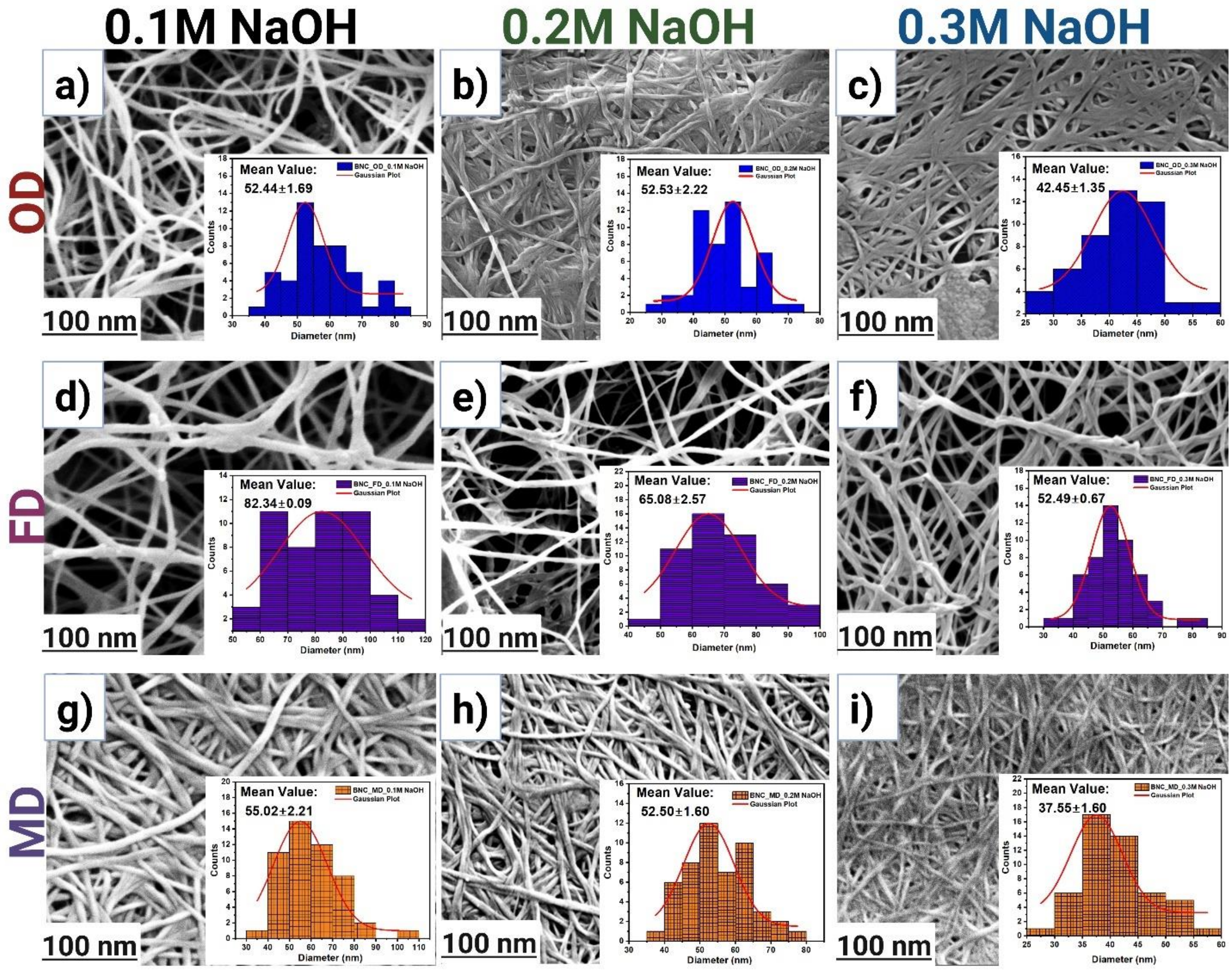

Figure S5. SEM images at 100nm scale of alkaline-treated dried nanofibers from bacterial nanocellulose at the following concentrations: (a,d,g) 0.1M NaOH; (b,e,h) 0.2M NaOH; and (c,f,i) 0.3M NaOH with inset SEM histogram wherein diameter was measured 50 times. The scanning electron micrographs were obtained using JEOL JSM-IT800 Schottky FESEM (Zeiss, Jena, Germany). Nanocellulose diameters were measured using ImageJ software (U.S. National Institutes of Health, Bethesda, Maryland, USA), 50 times per sample.

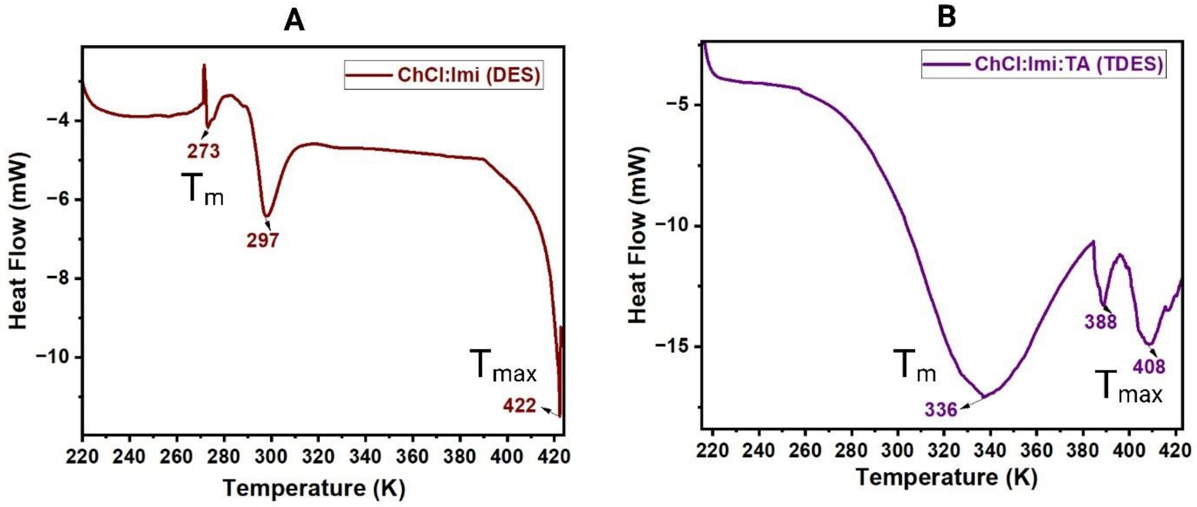

Figure. S6. Melting temperature of the Deep Eutectic solvent (A) and Ternary Deep Eutectic Solvent (B) using DSC.

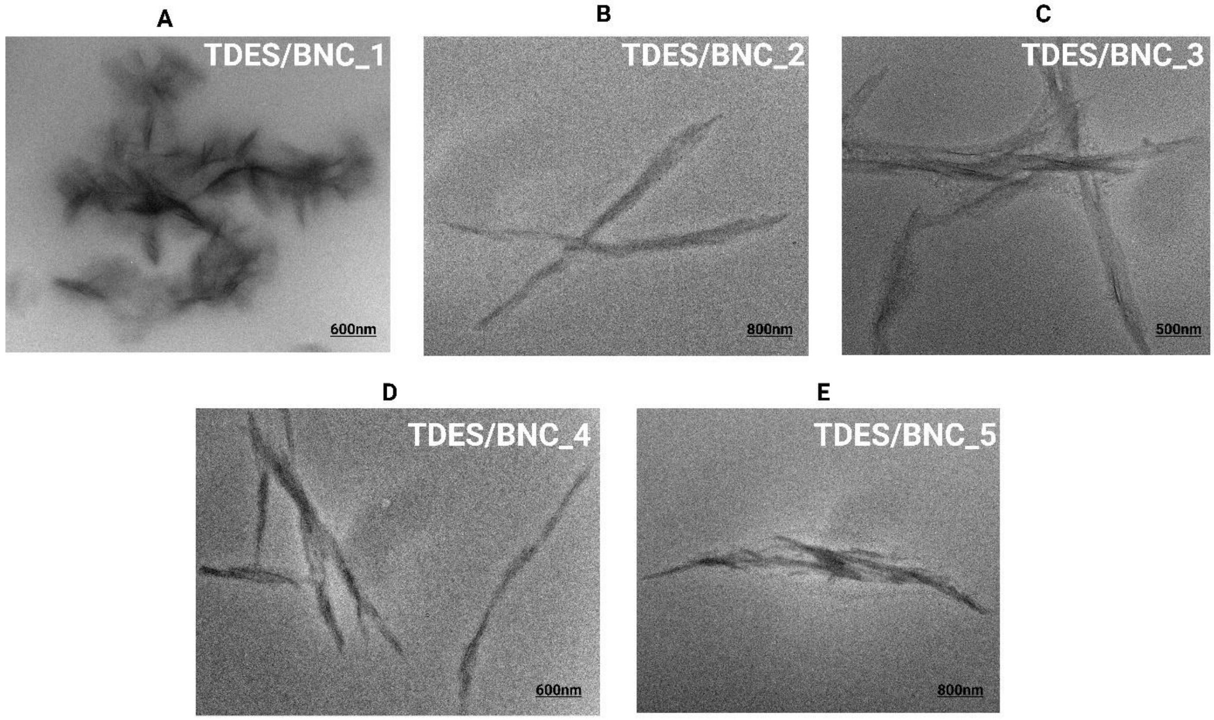

Figure. S7. TEM images showing BNC structure in TDES after dissolution.

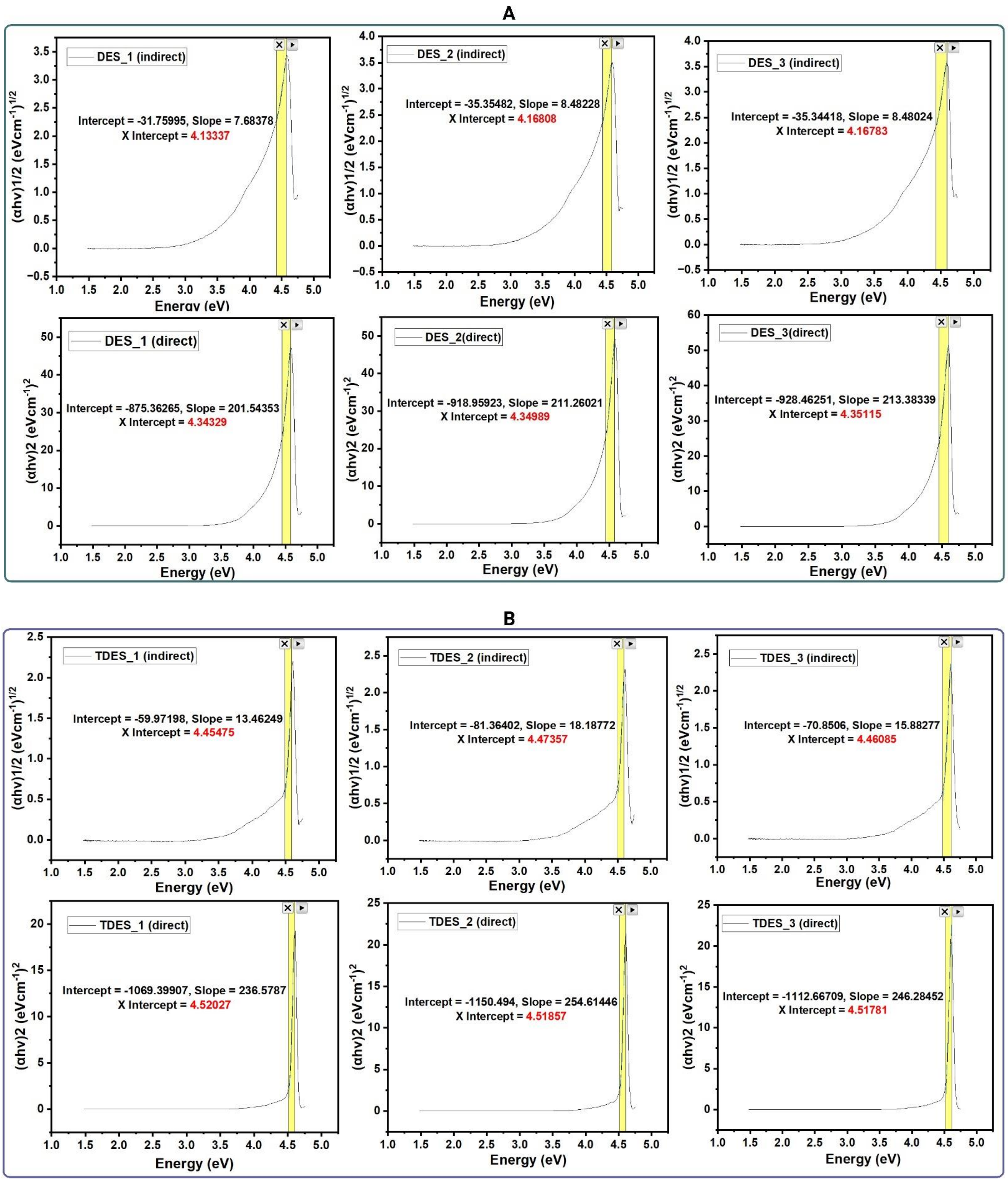
A
DES_1 (indirect)
Intercept = -31.75995, Slope = 7.68378
X Intercept = 4.13337
DES_2 (indirect)
Intercept = -35.35482, Slope = 8.48228
X Intercept = 4.16808
DES_3 (indirect)
Intercept = -35.34418, Slope = 8.48024
X Intercept = 4.16783
DES_1 (direct)
Intercept = -875.36265, Slope = 201.54353
X Intercept = 4.34329
DES_2(direct)
Intercept = -918.95923, Slope = 211.26021
X Intercept = 4.34989
DES_3(direct)
Intercept = -928.46251, Slope = 213.38339
X Intercept = 4.35115
(αhv)1/2 (eVcm^{-1})$^{1/2}$
(αhv)2 (eVcm^{-1})^2
Energy (eV)
B
TDES_1 (indirect)
Intercept = -59.97198, Slope = 13.46249
X Intercept = 4.45475
TDES_2 (indirect)
Intercept = -81.36402, Slope = 18.18772
X Intercept = 4.47357
TDES_3 (indirect)
Intercept = -70.8506, Slope = 15.88277
X Intercept = 4.46085
TDES_1 (direct)
Intercept = -1069.39907, Slope = 236.5787
X Intercept = 4.52027
TDES_2 (direct)
Intercept = -1150.494, Slope = 254.61446
X Intercept = 4.51857
TDES_3 (direct)
Intercept = -1112.66709, Slope = 246.28452
X Intercept = 4.51781
(αhv)1/2 (eVcm^{-1})$^{1/2}$
(αhv)2 (eVcm^{-1})^2
Energy (eV)

C

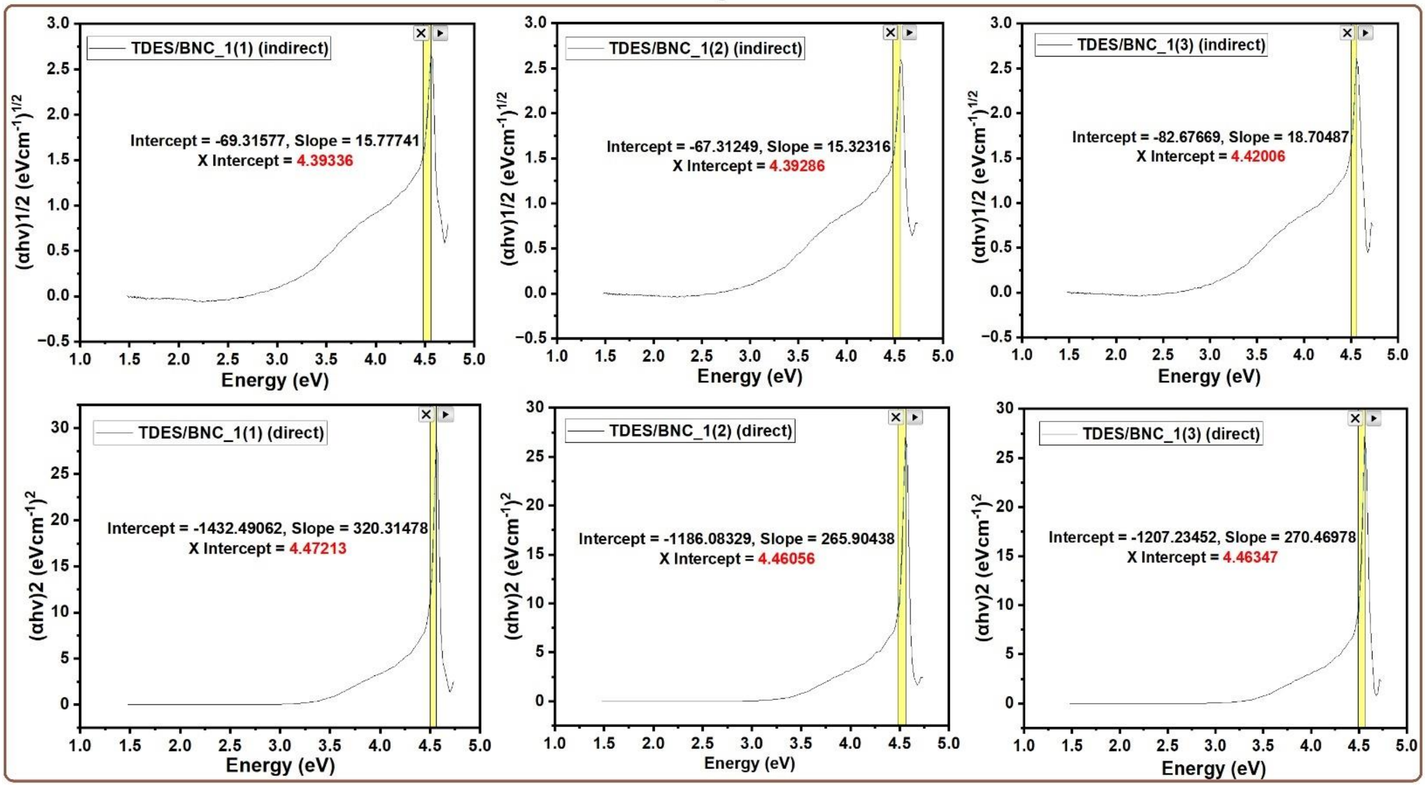
TDES/BNC_1(1) (indirect)
Intercept = -69.31577, Slope = 15.77741
X Intercept = 4.39336
TDES/BNC_1(2) (indirect)
Intercept = -67.31249, Slope = 15.32316
X Intercept = 4.39286
TDES/BNC_1(3) (indirect)
Intercept = -82.67669, Slope = 18.70487
X Intercept = 4.42006
TDES/BNC_1(1) (direct)
Intercept = -1432.49062, Slope = 320.31478
X Intercept = 4.47213
TDES/BNC_1(2) (direct)
Intercept = -1186.08329, Slope = 265.90438
X Intercept = 4.46056
TDES/BNC_1(3) (direct)
Intercept = -1207.23452, Slope = 270.46978
X Intercept = 4.46347
(αhv)1/2 (eVcm^{-1})$^{1/2}$
(αhv)2 (eVcm^{-1})^{2}
Energy (eV)

D

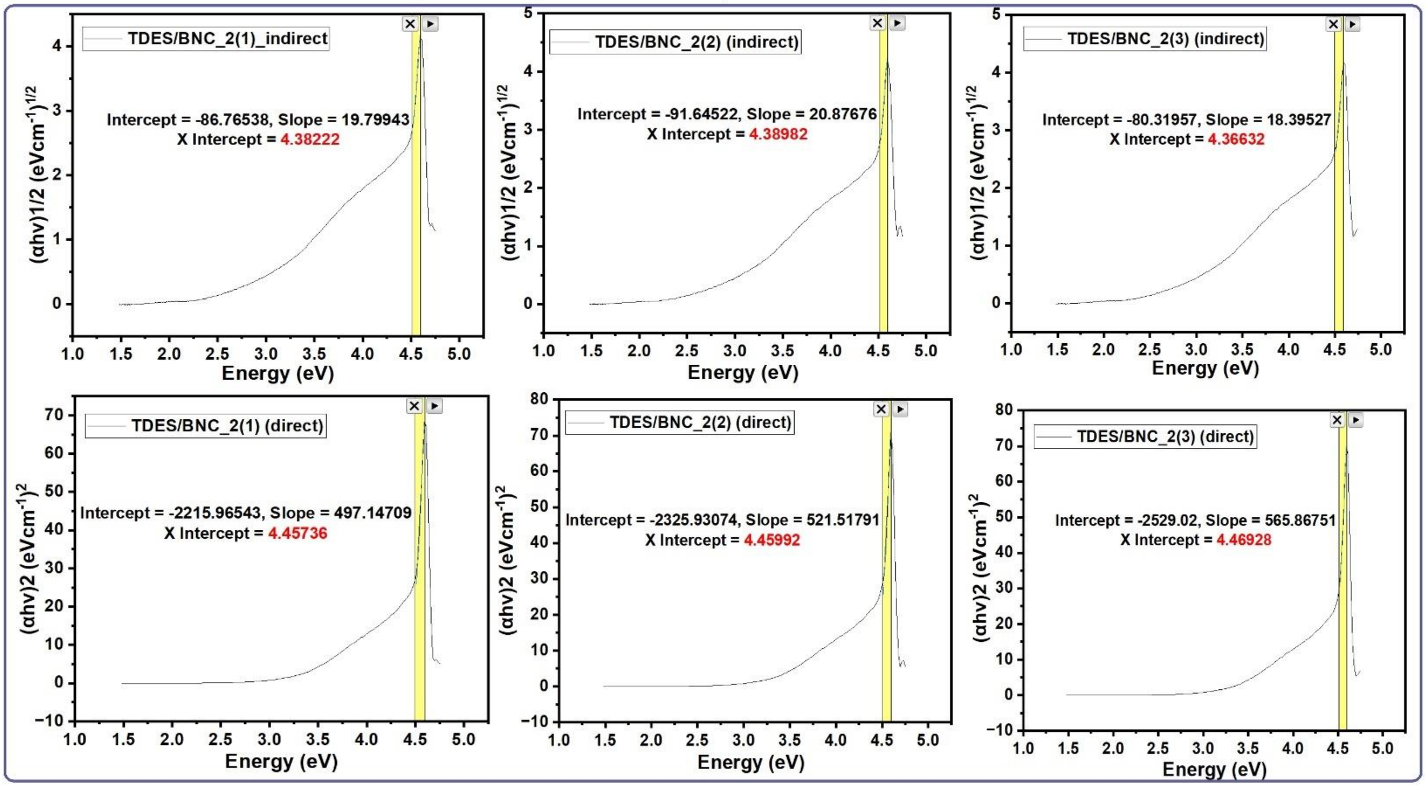
TDES/BNC_2(1)_indirect
Intercept = -86.76538, Slope = 19.79943
X Intercept = 4.38222
TDES/BNC_2(2) (indirect)
Intercept = -91.64522, Slope = 20.87676
X Intercept = 4.38982
TDES/BNC_2(3) (indirect)
Intercept = -80.31957, Slope = 18.39527
X Intercept = 4.36632
TDES/BNC_2(1) (direct)
Intercept = -2215.96543, Slope = 497.14709
X Intercept = 4.45736
TDES/BNC_2(2) (direct)
Intercept = -2325.93074, Slope = 521.51791
X Intercept = 4.45992
TDES/BNC_2(3) (direct)
Intercept = -2529.02, Slope = 565.86751
X Intercept = 4.46928
(αhv)1/2 (eVcm^{-1})$^{1/2}$
(αhv)2 (eVcm^{-1})^{2}
Energy (eV)

E

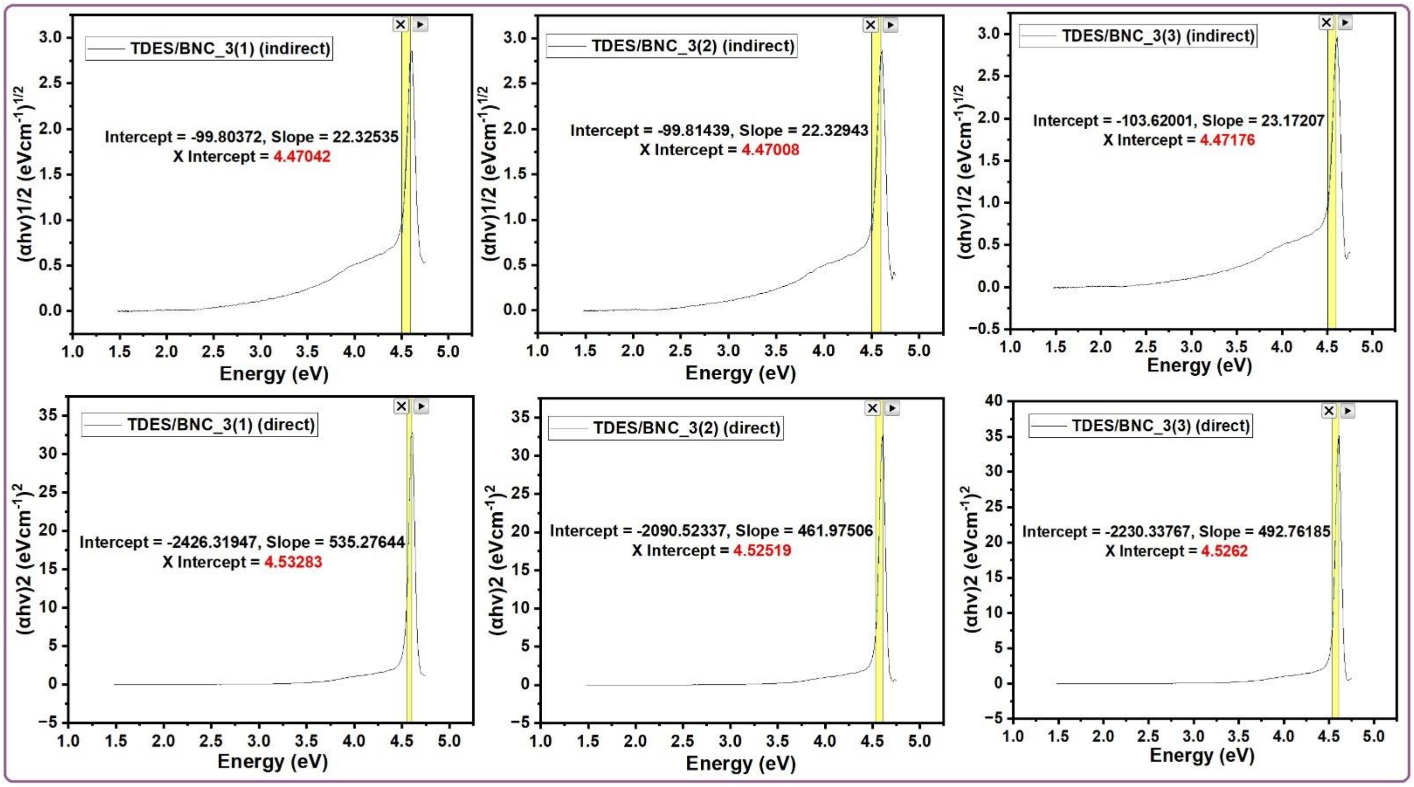
TDES/BNC_3(1) (indirect)
Intercept = -99.80372, Slope = 22.32535
X Intercept = 4.47042
TDES/BNC_3(2) (indirect)
Intercept = -99.81439, Slope = 22.32943
X Intercept = 4.47008
TDES/BNC_3(3) (indirect)
Intercept = -103.62001, Slope = 23.17207
X Intercept = 4.47176
TDES/BNC_3(1) (direct)
Intercept = -2426.31947, Slope = 535.27644
X Intercept = 4.53283
TDES/BNC_3(2) (direct)
Intercept = -2090.52337, Slope = 461.97506
X Intercept = 4.52519
TDES/BNC_3(3) (direct)
Intercept = -2230.33767, Slope = 492.76185
X Intercept = 4.5262
(αhv)1/2 (eVcm^{-1})$^{1/2}$
(αhv)2 (eVcm^{-1})^2
Energy (eV)

F

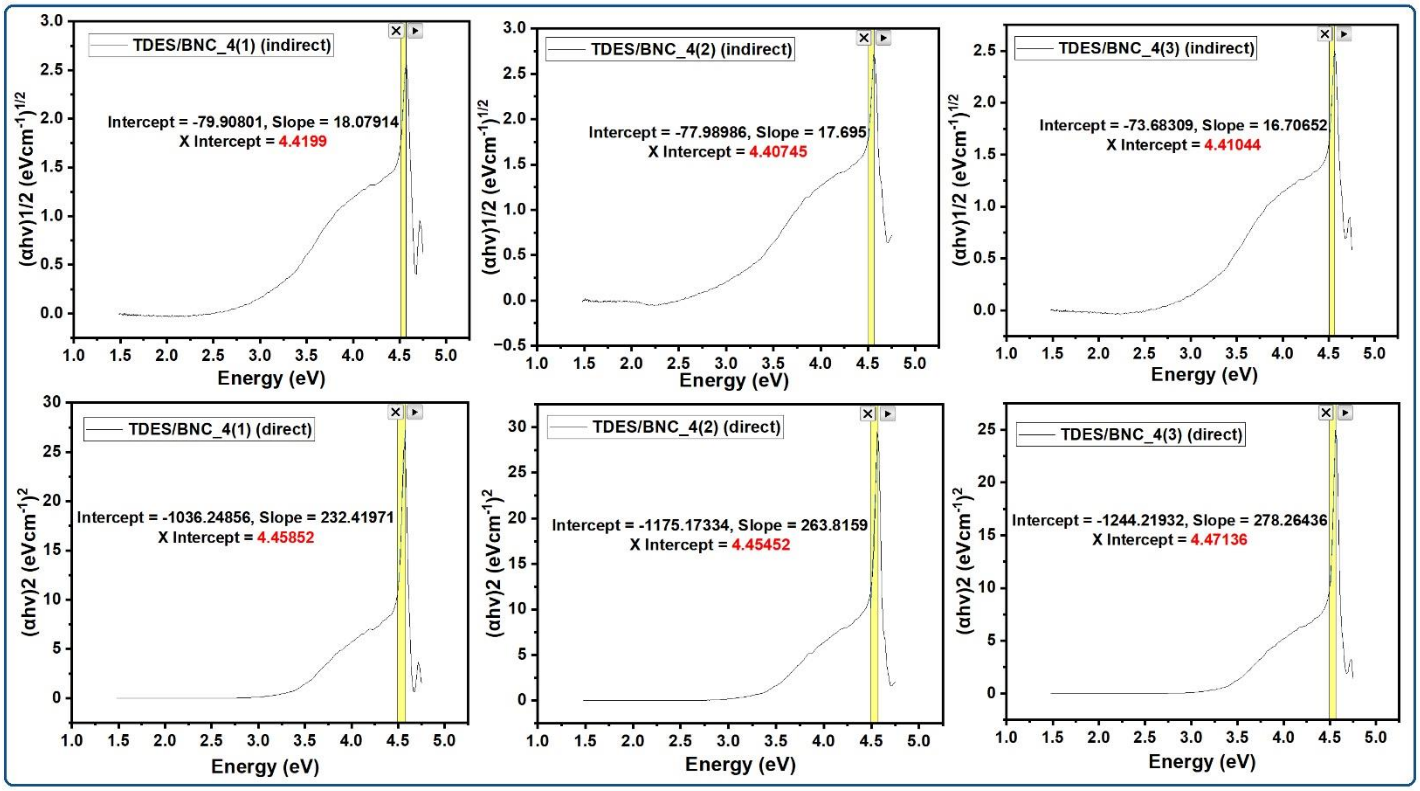
TDES/BNC_4(1) (indirect)
Intercept = -79.90801, Slope = 18.07914
X Intercept = 4.4199
TDES/BNC_4(2) (indirect)
Intercept = -77.98986, Slope = 17.695
X Intercept = 4.40745
TDES/BNC_4(3) (indirect)
Intercept = -73.68309, Slope = 16.70652
X Intercept = 4.41044
TDES/BNC_4(1) (direct)
Intercept = -1036.24856, Slope = 232.41971
X Intercept = 4.45852
TDES/BNC_4(2) (direct)
Intercept = -1175.17334, Slope = 263.8159
X Intercept = 4.45452
TDES/BNC_4(3) (direct)
Intercept = -1244.21932, Slope = 278.26436
X Intercept = 4.47136
(αhv)1/2 (eVcm^{-1})$^{1/2}$
(αhv)2 (eVcm^{-1})^2
Energy (eV)

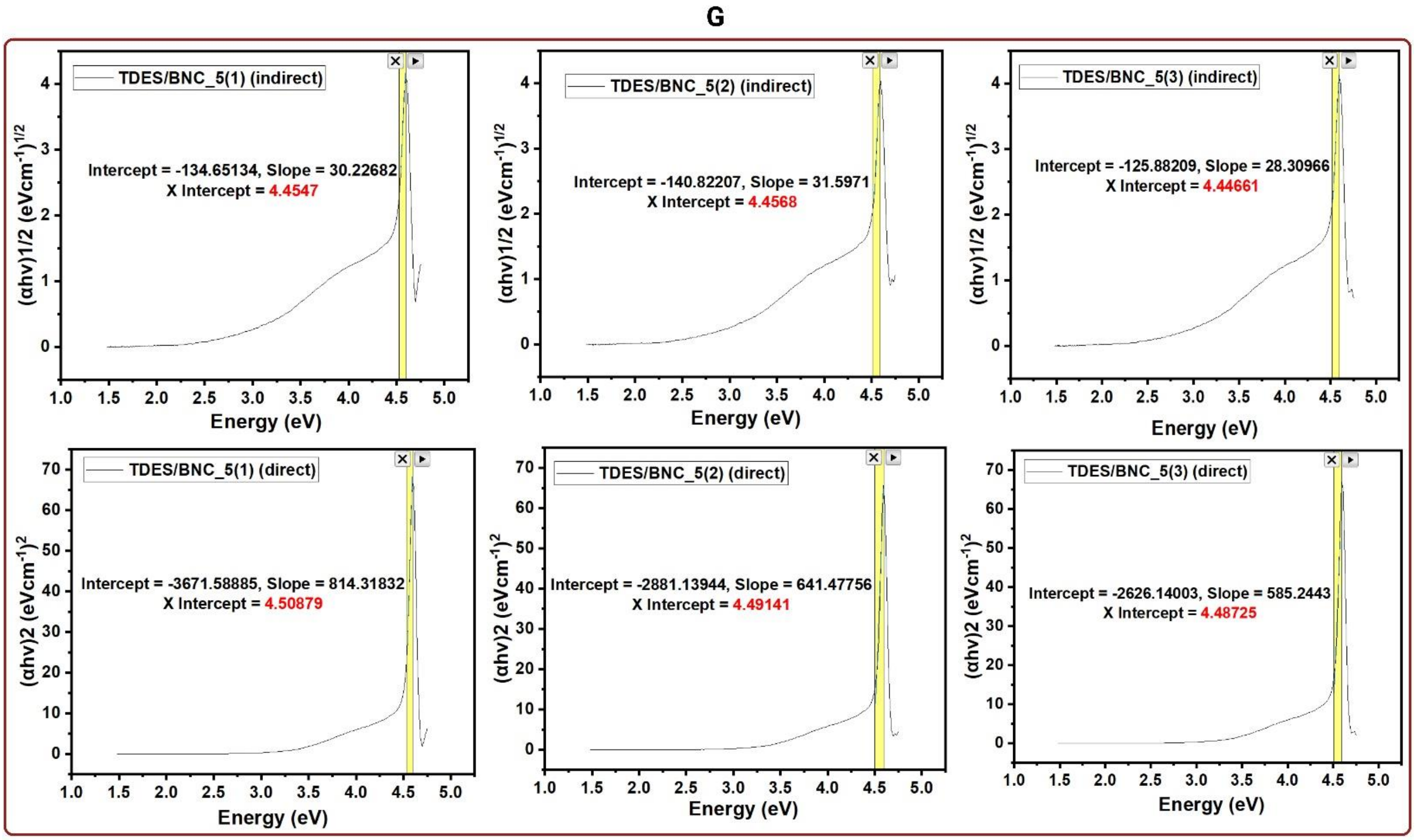

Figure. S8. Measured Indirect and direct band in triplicate samples for DES (A); TDES (B); TDES/BNC_1 (C); TDES/BNC_2 (D); TDES/BNC_3 (E); TDES/BNC_4 (F); TDES/BNC_5 (G).

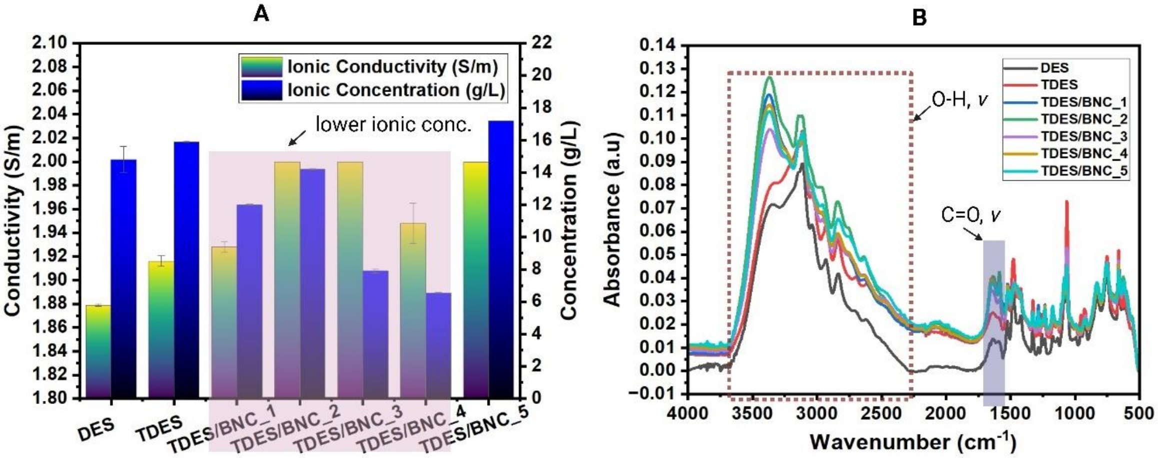

Figure. S9. Ionic Conductivity and Ionic Concentration (A); ATR-FTIR spectrum (B).

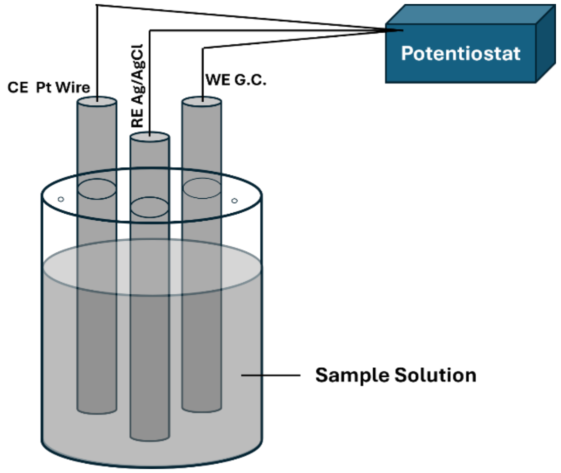

Figure. S10. Cyclic Voltammetry set up used for DES, TDES, and TDES/BNC samples solution.

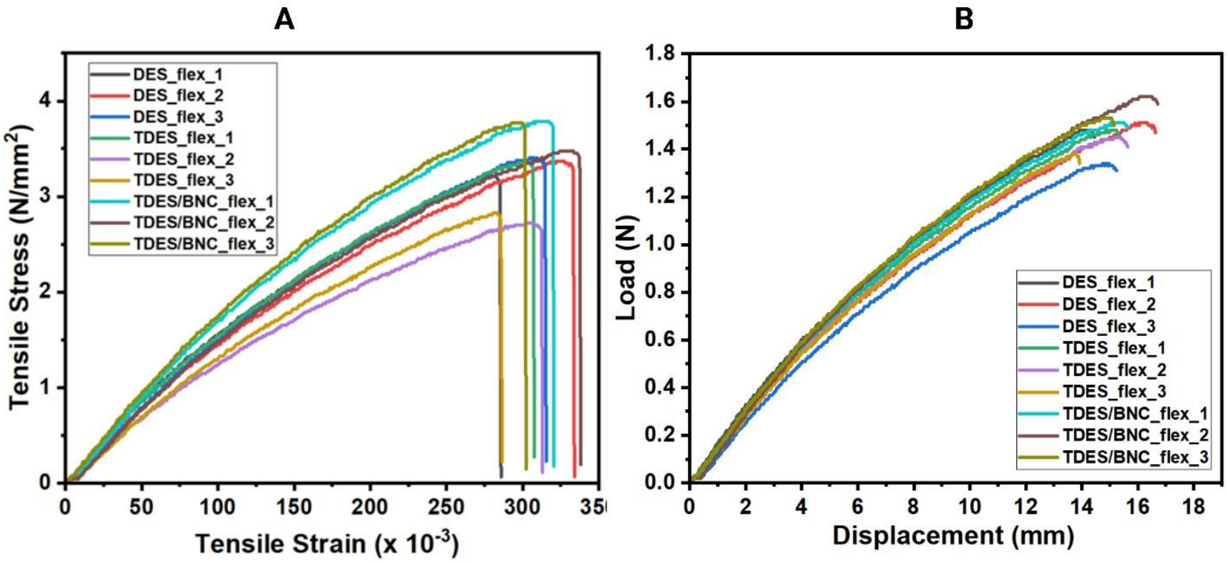

Figure. S11. Stress-Strain curve (A) and Load vs Displacement (B) of flexible materials

References:

1. Muzio, S.D., Russina, O., Mastrippolito, D., Benassi, P., Rossi, L., Paolone, A., Ramondo, F. Mixtures of choline chloride and tetrabutylammonium bromide with imidazole as examples of deep eutectic solvents: their structure by theoretical and experimental investigation. *Journal of Molecular Liquids,* **2022,** 352, 118427.
2. Mathworks, "Interactionplot: Interaction plot for grouped data," Link: Interaction plot for grouped data - MATLAB interactionplot (mathworks.com). Accessed: June 26, 2024.
3. Agar Foil. Link: https://class.textile-academy.org/2020/loes.bogers/files/recipes/agarfoil/. Accessed: March 16, 2025
4. Yamamoto, H., Horii, F., Hirai, A. "In situ crystallization of bacterial cellulose II. Influences of different polymeric additives on the formation of celluloses Iα and Iβ at the early stage of incubation," *Cellulose*, **1996,** 3, 229-242.
5. Grube, M., Shvirksts, K., Denina, I., Ruklisa, M., Semjonovs, P. "Fourier-transform infrared spectroscopic analyses of cellulose from different bacterial cultivations using microspectroscopy and a high-throughput screening device," *Vibrational Spectroscopy*, **2016,** 84, 53-57.
6. A. Patterson. "The Scherrer formula for X-ray particle size determination," *Phy. Rev.* **1939,** 10, 978.
7. Segal, L., Creely, J.J., Martin, A.E., Conrad, C.M. "An Empirical Method for Estimating the Degree of Crystallinity of Native Cellulose Using the X-Ray Diffractometer," *Textile Research Journal*, **1959,** 29, 786-794.
8. Atykyan, N., Revin, V., Shutova, V. (2020) "Raman and FT-IR Spectroscopy investigation the cellulose structural differences from bacteria *Gluconacetobacter sucrofermentans* during the different regimes of cultivation on a molasses media," *AMB Express,* 10, 84. https://doi.org/10.1186/s13568-020-01020-8.
9. Tauc, J., Grigorovici, R., Vancu, R. (1966). "Optical Properties and Electronic Structure of Amorphous Germanium," *Physica Status Solidi (b)*, 15, pp. 627-637. https://doi.org/10.1002/pssb.19660150224
10. Mu, S., Yang, Y. "Spectral Characteristics of Polyaniline Nanostructures Synthesized by Using Cyclic Voltammetry at Different Scan Rates," 112, 37, 11558-11563. 10.1021/jp8051517
11. Naveen, C., Muthuvinayagam, M., Alrashidi, K.A, Mohammad, S., Vigneshwaran, S., Arunachalam, S., Isa, M.I. (2024). "Investigations on electrical, electrochemical, and thermal properties of gelatine-based novel biopolymer electrolytes for energy storage applications," *Ionics*, 30, pp. 6097-6111. https://doi.org/10.1007/s11581-024-05750-8
12. The Voltera Team, Electronics 101: Resistance, Resistivity, and Sheet Resistance. Link: https://www.voltera.io/blog/resistance-resistivity-and-sheet-resistance. Accessed: August 26, 2025.
13. LibreText Physics: 9.4: Resistivity and Resistance. Link: https://phys.libretexts.org/Bookshelves/University_Physics/University_Physics_(OpenStax)/University_Physics_II_-_Thermodynamics_Electricity_and_Magnetism_(OpenStax)/09%3A_Current_and_Resistance/9.04%3A_Resistivity_and_Resistance. Accessed: August 26, 2025.
14. ISO 37: Rubber, vulcanized or thermoplastic — Determination of tensile stress-strain properties. Link: https://www.mecmesin.com/standard/iso-37. Accessed: August 26, 2025.
15. Plackett, R. L. & Burman, J. P. Te design of optimum multifactorial experiments. Biometrika 33, 305–325 (1946).
16. Tjur, T., Bailey, R. A., Speed, T. P. & Wynn, H. P. Analysis of variance models in orthogonal designs: Reply to discussion. Int. Stat. Rev./Revue Internationale de Statistique 1, 52 (1984).

17. Mathworks, “Interactionplot: Interaction plot for grouped data,” Link: Interaction plot for grouped data - MATLAB interactionplot (mathworks.com). Accessed: June 26, 2024.
18. Ortega, J.M.P., Ochoa-Barragan, R., Ramirez-Marquez, Cesar.,(2024) “Optimization Using the Software MATLAB,” *Optimization of Chemical Processes*. Springer, Cham. https://doi.org/10.1007/978-3-031-57270-8_13.
